# Topical tapinarof (benvitimod) for papulopustular rosacea: two cases^؆^

**DOI:** 10.1016/j.abd.2026.501344

**Published:** 2026-04-22

**Authors:** Lanqi Wang, Sitong Li, Jiacheng Lin, Qiong Wu, Qiang Ju

**Affiliations:** Department of Dermatology, School of Medicine, Renji Hospital, Shanghai Jiao Tong University, Shanghai, PR, China

Dear Editor,

Rosacea is a chronic inflammatory disorder that primarily affects the central face or eyes, characterized by flushing, persistent erythema, telangiectasia, papules, pustules, edema, and phymatous changes, with ocular involvement manifesting as blepharitis, conjunctival hyperemia, and dry eye symptoms. Current first-line topicals (metronidazole, azelaic acid, and ivermectin) offer variable efficacy and tolerability, while systemic agents may cause dysbiosis, dizziness, photosensitivity, and teratogenicity in selected populations.^1^, ^2^ Tapinarof (also known as benvitimod) is a nonsteroidal small-molecule agonist of the Aryl hydrocarbon Receptor (AhR) with anti-inflammatory, barrier-restoring, and sebo-suppressive effects.^3^ It is approved for plaque psoriasis and has shown benefit in atopic dermatitis.^4^ To our knowledge, tapinarof has not been reported for the treatment of rosacea. We presented two patients with Papulopustular Rosacea (PPR) treated with topical 1% tapinarof cream. Disease severity was assessed by Investigator Global Assessment (IGA; 0‒4) by the treating dermatologist and patient-reported Dermatology Life Quality Index (DLQI; 0‒30).

## Case 1

**Fig. 1:**
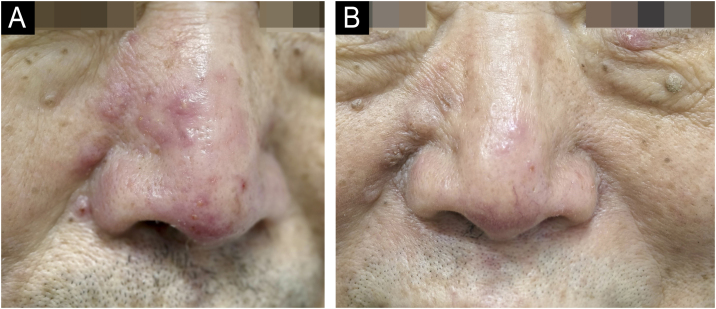
Clinical images of Patient 1 with papulopustular rosacea. (A) Nasal erythematous papules at baseline. (B) After 4-weeks of once-daily 1% tapinarof cream.

A 66-year-old man presented with a 2-month history of persistent nasal erythema accompanied by several inflammatory papules and mild discomfort. Previous therapy with 0.75% metronidazole gel had yielded minimal improvement. Physical examination revealed centrofacial erythema with discrete erythematous papules and seborrhoea. Topical 1% tapinarof cream was applied once nightly. By week 4, erythema and papules were almost cleared (IGA 2 to 1), the DLQI improved from 5 to 0, and seborrhoea was reduced (Fig. 1A‒B). Then the tapinarof was discontinued. No local or systemic adverse events occurred. Without maintenance therapy, the therapeutic response was maintained at the 6-month mark.

## Case 2

**Fig. 2:**
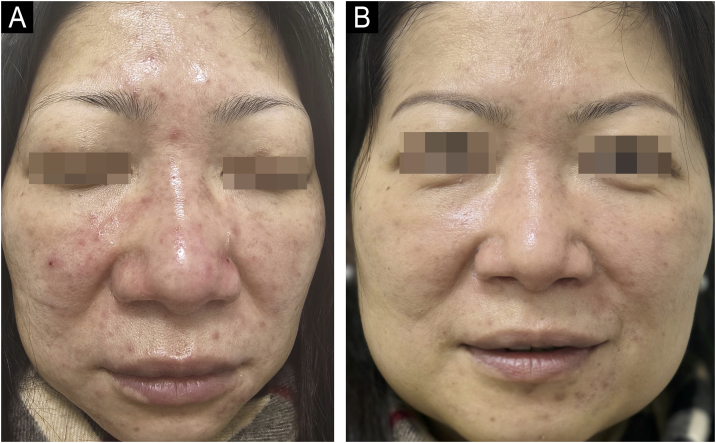
Clinical images of Patient 2 with papulopustular rosacea. (A) Centrofacial persistent erythema with papules and pustules at baseline. (B) After 8-weeks of treatment.

A 48-year-old woman reported a 5-year history of centrofacial persistent erythema, papules, and papulopustules accompanied by excessive facial sebum. She declined systemic therapy. Serological screening for autoimmune connective-tissue disease (antinuclear, anti-extractable nuclear antigen, anti-dsDNA, and anti-mitochondrial antibodies) was negative. Baseline IGA was 3, and DLQI was 14. Treatment with 1% tapinarof cream, applied nightly for 4-weeks and then every other night for a further 4-weeks, was initiated. The lesion count and erythema were substantially reduced (IGA 3 to 1), and sebum production diminished; DLQI improved to 3 (Fig. 2A‒B). The patient received no maintenance therapy. No adverse effects were observed. The therapeutic response persisted throughout 4-months of follow-up. The patient reported a mild relapse at 4-months post-discontinuation, characterized by faint central facial erythema and fewer than five scattered papulopustular lesions; these lesions improved following self-initiated tapinarof.

Rosacea has different manifestations. PPR is characterized by a centrofacial eruption of multiple papules and/or papulopustules with persistent centrofacial erythema.^1^, ^2^ These two cases demonstrated that 1% tapinarof cream, applied once nightly, rapidly attenuated papulopustular lesions and reduced seborrhoea, with sustained remission over 3-months. The improvement in DLQI underscored meaningful patient-reported benefit. No cutaneous irritation, contact sensitization, or systemic toxicity was observed, supporting favorable tolerability.

The observed efficacy aligns with mechanistic insights. Demodex infestation rates are significantly higher in the rosacea group than in healthy controls.^1^, ^2^ Demodex mites may activate Toll-Like Receptor-2 (TLR2). TLR2-dominated innate immunity contributes to the development of rosacea. When TLR2 is activated, keratinocytes produce proinflammatory cytokines and chemokines. Skin samples from patients with rosacea exhibit increased expression of proinflammatory cytokines such as IL-8, IL-1β, and TNF-α. IL-8 leads to the chemotaxis of neutrophils in the skin. IL-1β and TNF-α promote further inflammatory reactions. TLR2 helps to increase the expression of KLK5, which is essential for activation of LL37.^5^ Adaptive immune system activation, shown by the presence of T-Helper 1 (TH1) and T-Helper 17 (TH17) cells with their corresponding immune mediators in skin lesions of rosacea, results in increased inflammation and further immune activation. An upregulation of IFN-γ and IL-17A in rosacea-affected skin was also identified. IL-17 has been shown to induce angiogenesis through VEGF and affect the expression of LL-37 in human keratinocytes.^1^, ^2^ AhR activation by tapinarof suppresses TLR2-mediated innate responses, curtails Th17 cytokine production, and down-regulates sebaceous lipogenesis, thereby mitigating both inflammatory lesions and the lipid-rich milieu that favors Demodex proliferation.^3^, ^5^, ^6^, ^7^ However, the precise mechanisms underlying tapinarof’s effect in rosacea require further investigation.

Our study suggests that topical tapinarof might be a potentially effective alternative for PPR. The small sample size and short follow-up period are limitations of this study. Controlled studies are warranted to confirm these preliminary findings and to establish optimal dosing frequency.

## ORCID ID

Lanqi Wang: 0000-0002-9211-5600

Jiacheng Lin: 0009-0001-8555-770X

Qiong Wu: 0000-0002-8586-6860

Qiang Ju: 0000-0002-0251-1598

## Financial support

This study was supported by the National Natural Science Foundation of China (No. 82173434).

## Authors' contributions

Lanqi Wang*: Preparation and writing of the manuscript; data collection, analysis and interpretation; approval of the final version of the manuscript.

Sitong Li*: Preparation and writing of the manuscript; data collection, analysis and interpretation; approval of the final version of the manuscript.

Jiacheng Lin: Preparation and writing of the manuscript; manuscript critical review; approval of the final version of the manuscript.

Qiong Wu: Preparation and writing of the manuscript; manuscript critical review; approval of the final version of the manuscript.

Qiang Ju: Effective participation in research orientation; study conception and planning; data analysis and interpretation; manuscript critical review; approval of the final version of the manuscript.

*These authors contributed equally to this work and share first authorship.

## Research data availability

Does not apply.

## Conflicts of interest

None declared.
